# Machine Learning-Based Decision-Making in Geriatrics: Aging Phenotype Calculator and Survival Prognosis

**DOI:** 10.14336/AD.2024.0120

**Published:** 2024-01-20

**Authors:** Aleksandra Mamchur, Natalia Sharashkina, Veronika Erema, Daria Kashtanova, Mikhail Ivanov, Maria Bruttan, Elena Zelenova, Eva Shelly, Valentina Ostapenko, Irina Dzhumaniiazova, Lorena Matkava, Vladimir Yudin, Anna Akopyan, Irina Strazhesko, Lilit Maytesyan, Irina Tarasova, Olga Beloshevskaya, Anton Keskinov, Sergey Kraevoy, Olga Tkacheva, Sergey Yudin

**Affiliations:** ^1^Centre for Strategic Planning and Management of Biomedical Health Risks, Federal Medical Biological Agency, Moscow, Russia.; ^2^Russian Gerontology Research and Clinical Center, Pirogov Russian National Research Medical University, Moscow, Russia.

**Keywords:** long-living adults, longevity, aging phenotypes, patient stratification, machine learning, survival analysis

## Abstract

Aging is a natural process with varying effects. As we grow older, our bodies become more susceptible to aging-associated diseases. These diseases, individually or collectively, lead to the formation of distinct aging phenotypes. Identifying these aging phenotypes and understanding the complex interplay between coexistent diseases would facilitate more personalized patient management, a better prognosis, and a prolonged lifespan. Many studies distinguish between successful aging and frailty. However, this simple distinction fails to reflect the diversity of underlying causes. In this study, we sought to establish the underlying causes of frailty and determine the patterns in which these causes converge to form aging phenotypes. We conducted a comprehensive geriatric examination, cognitive assessment, and survival analysis of 2,688 long-living adults (median age = 92 years). The obtained data were clustered and used as input data for the Aging Phenotype Calculator, a multiclass classification model validated on an independent dataset of 96 older adults. The accuracy of the model was assessed using the receiver operating characteristic curve and the area under the curve. Additionally, we analyzed socioeconomic factors that could contribute to specific aging patterns. We identified five aging phenotypes: non-frailty, multimorbid frailty, metabolic frailty, cognitive frailty, and functional frailty. For each phenotype, we determined the underlying diseases and conditions and assessed the survival rate. Additionally, we provided management recommendations for each of the five phenotypes based on their distinct features and associated challenges. The identified aging phenotypes may facilitate better-informed decision-making. The Aging Phenotype Calculator (ROC AUC = 92%) may greatly assist geriatricians in patient management.

## INTRODUCTION

Modern medicine has made great strides in extending life expectancy and mitigating the effects of chronic illnesses. However, the impact of aging varies from person to person. While some nonagenarians and centenarians maintain physical and cognitive health, others succumb to aging-associated diseases. Consequently, there is a growing recognition of the importance of patient stratification that enables precision therapy, personalized medicine, and, ultimately, improved quality of life. The term ‘aging phenotype’ encompasses both frailty and non-frailty phenotypes. In people with the frailty phenotype, it is crucial to precisely identify the specific frailty phenotype and determine the underlying conditions.

Passarino et al. examined aging phenotypes in two groups of individuals: S1 (n = 252; age = 65-85) and S2 (n = 117; age = 90+). They identified three aging phenotypes in S2 (non-frail, intermediate, and frail) and only two in S1 (frail and very frail) [1]. Marcucci et al. identified four clusters in 2,841 patients aged 65+ years [2]: the healthiest individuals; individuals with multiple chronic conditions; functionally independent women with osteoporosis and arthritis; and functionally dependent older patients with cognitive impairment. We have also previously demonstrated that aging patterns vary greatly, with some older adults aging successfully and others aging unsuccessfully or even extremely unsuccessfully [3].

The frailty phenotype, also known as unsuccessful aging, has been found in all the above studies. Older patients are more prone to comorbidities, dependency, and poor outcomes [4]. However, the term frailty is not specific or indicative of the underlying causes of health conditions and functional decline. In an effort to address this issue, Liu et al. studied frailty in 1,008 participants aged 50+ years. They identified three mobility-based frailty sub-phenotypes: non-mobility type (loss and exhaustion); mobility type (slowness and weakness); and low physical activity. Participants with the mobility type had poorer body composition, bone health, cognitive function, survival rate, and overall outcomes [5].

In this study, we sought to challenge the binary approach to phenotyping frailty and expand our understanding of how the underlying health conditions form specific frailty phenotypes. Based on the existing research on frailty, including our previous findings, we hypothesized that a cluster analysis of extensive data on the underlying health conditions and socioeconomic backgrounds could reveal distinct aging phenotypes, while a one-year follow-up would allow us to assess the mortality rate. For this purpose, we conducted a comprehensive geriatric assessment of the participants for 15 geriatric syndromes and examined their medical histories and socioeconomic background. To test our hypothesis, we conducted two-step data clustering and developed a personalized Aging Phenotype Calculator (APC), which is a machine learning (ML)-based multiclass classification model. The generated data clusters were used as input. The calculator produced five aging phenotypes. To validate our findings, we tested the model on an independent sample of older adults and used the area under the receiver operating characteristic curve, or ROC AUC, as a standard measure of accuracy. We obtained a ROC AUC of 92%.

We developed the APC as a decision-making tool to aid geriatricians in providing optimal, timely, and individualized care to their patients. For this purpose, it also integrates phenotype-specific recommendations for better health outcomes. The APC is based on the analytic techniques and extensive patient data described below. However, as a new clinical tool, the APC needs a closer evaluation in real-world clinical settings to further verify the claimed benefits and improve its accuracy.

## MATERIALS AND METHODS

### Participant recruitment and examination

We analyzed data from 2,688 individuals aged 90 years and older from the Central Federal District of Russia, recruited with the assistance of social and geriatric services of Moscow and the Moscow Region between 2019 and 2023. We examined their medical histories, socioeconomic backgrounds, and the risk of chronic diseases. All participants provided informed consent for the collection of blood and biomaterial samples, as well as for two visits by a physician.

The participants underwent a comprehensive geriatric assessment for the following 15 geriatric syndromes, in accordance with the clinical recommendations of the Ministry of Health of the Russian Federation [https://sudact.ru/law/klinicheskie-rekomendatsii-starcheskaia-asteniia-utv-minzdravom-rossii/] (the methods of assessment are indicated in brackets):

- Frailty syndrome (the Short Physical Performance Battery);
- Cognitive impairment (the Mini-Mental State Examination (MMSE) and clock-drawing test);
- Frontal lobe dysfunction (the Frontal Assessment Battery (FAB) test);
- Chronic pain;
- Anxiety disorder;
- Risk of falls;
- Sensory deficit;
- Depression (the Five-Item Geriatric Depression Scale (GDS-5));
- Sarcopenia (the SARC-F questionnaire (strength, assistance walking, rise from a chair, climb stairs, and falls) and hand-held dynamometry);
- Risk of malnutrition (the Mini Nutritional Assessment (MNA-scale));
- Fecal or urinary incontinence (self-reported);
- Dependence in ADL (activity of daily living) (the Barthel index);
- Dependence in IADL (instrumental activity of daily living) (the Lawton scale);
- Polypharmacy, defined as the simultaneous administration of five or more medications;
- Orthostatic hypotension (standard diagnostic procedure) [www.mayoclinic.org/diseases-conditions/orthostatic-hypotension/diagnosis-treatment/drc-20352553].

In our previous study [3], we provided a detailed diagram of the above comprehensive geriatric assessment. For the one-year follow-up, we inquired about the participants’ health condition, health concerns, if any, in the past year, and, where appropriate, death and its causes. This information was obtained either through phone interviews or the examination of the participants’ medical records from outpatient or inpatient facilities. The survival data was available for 962. This data was used to create exponential curves for the survival analysis.

### Clustering and statistical analysis

**Figure 1.**
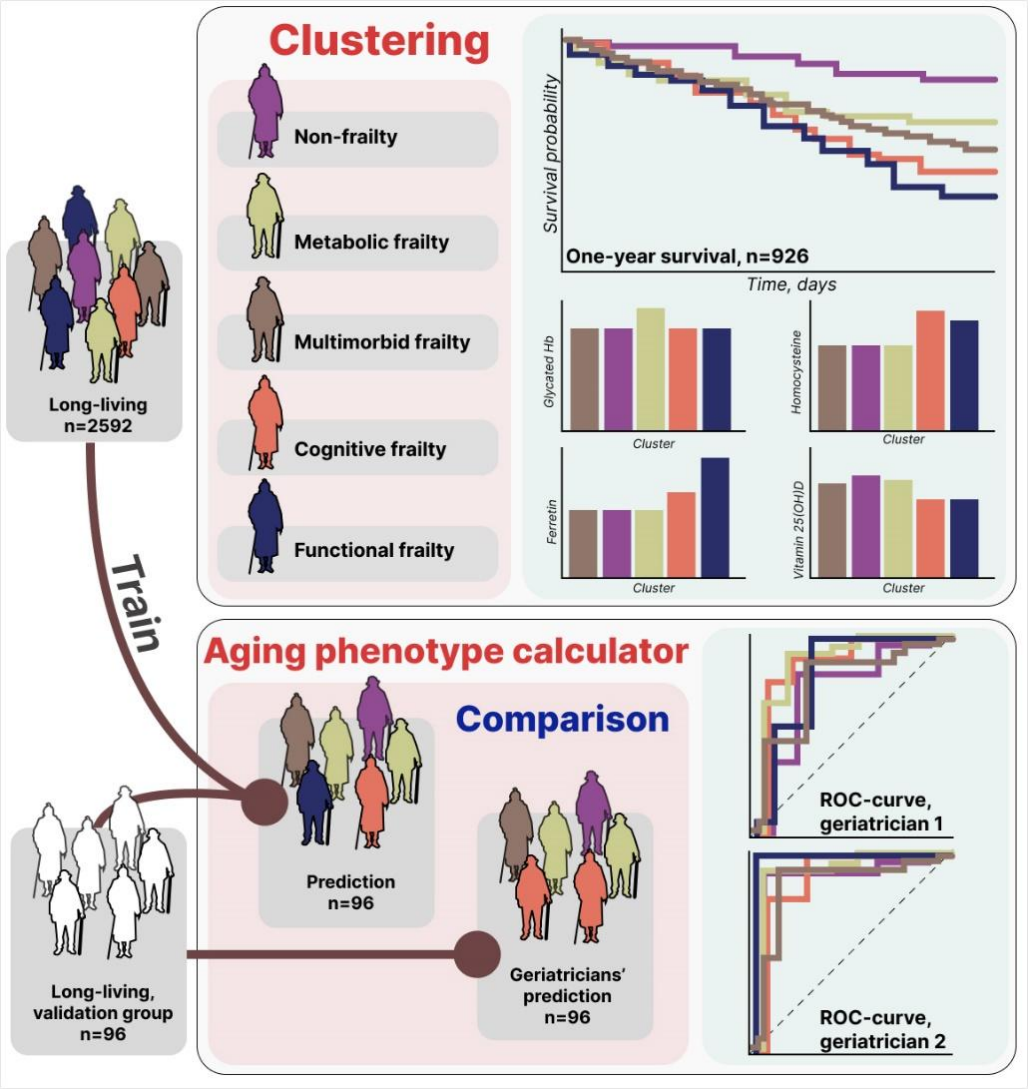
The research designs.

We clustered and analyzed the data from 2,592 participants with morbidities associated with cardiovascular diseases (CVDs), diabetes mellitus (DM), chronic obstructive pulmonary disease (COPD), cancer, and polypharmacy in two steps using Scikit-Learn's [6] Agglomerative Hierarchical Clustering in Python 3.9.12. We factored in BMI, right handgrip strength, dementia, frontal lobe dysfunction, physical performance, ADL, IADL, and depression (Fig 1).

First, we clustered the data based on all the aforementioned parameters. Then, we chose the larger cluster and divided it into four smaller clusters based on CVDs, DM, COPD, cancer, dementia (mild to moderate; MMSE <20), and frailty (SPPB <8).

We compared the five clusters using the Mann-Whitney U test and the Kruskal-Wallis test for continuous variables and the chi-squared test and Fisher's exact test for categorical variables. The distribution of the quantitative variables was non-normal across all groups. Therefore, we used nonparametric tests for their comparison.

To address the multiple testing problem, we applied the Bonferroni correction to all p-values. The significant threshold was 0.05. To simultaneously compare the five clusters, we conducted a total of 82 Kruskal-Wallis tests and chi-squared tests. Only significant associations are shown in Table 1 and Supplementary Table 1. For pairwise comparisons of the clusters, we conducted 440 tests (Supplementary Tables S2, S3, and S5). The tables show the results of the analysis of 44 variables across 10 pairs of clusters.

To test the associations between aging phenotypes and genetic, socioeconomic, and other risk factors, we carried out 40 iterations of logistic regression (five aging phenotypes, two associations with the APOE gene variants, and six socioeconomic factors). We used the aging phenotype as the target variable and the assessed factors, sex, and age as predictors and applied the Bonferroni correction to all obtained p-values. The tables below show adjusted p-values.

### One-year survival analysis

The one-year survival rate in 926 participants was assessed using *scikit-survival* 0.20.0 in Python 3.9.12. The Kaplan-Meier curves were compared using the sksurv.compare.compare_survival function.

To achieve the most accurate approximation, the curves were fitted using *numpy.polyfit* and the following equation:

$$y=a∙e^{b∙x}$$ (1)

The resulting curves were extrapolated up to 5,650 days. The median survival rate for each phenotype was evaluated based on the extrapolation results.

### Development of the Aging Phenotype Calculator

The Aging Phenotype Calculator (APC) is a machine learning-based multiclass classification model that was developed using a Support Vector Machine (SVM) from the scikit-learn library. As input for the ACP, we used the clustered data split into a training set (80%) and a test set (20%) and the following predictors: CVDs, DM, COPD, cancer, polypharmacy, BMI, right hand grip strength, and scores from MMSE, FAB, SPPB, ADL, IADL, and GDS-5. All predictors were standardized using the StandardScaler tool from the scikit-learn library. To assess the accuracy of the classification model, we constructed a receiver operating characteristic (ROC) curve using the one-vs-rest (OvR) strategy and calculated the area under the curve (AUC).

**Table 1:** Geriatric assessment results and health indicators in each cluster.

Cluster	0	1	2	3	4	p-value (Kruskal test)
Phenotype	Multimorbid frailty (n=1602)	Non-frailty (n=309)	Metabolic frailty (n=272)	Cognitive frailty (n=234)	Functional frailty (n=175)	
**Scale**	Me [Q1-Q3]	Me [Q1-Q3]	Me [Q1-Q3]	Me [Q1-Q3]	Me [Q1-Q3]	
**MMSE**	24.00 [22.00 - 27.00]	26.00 [24.00 - 28.00]	24.00 [20.00 - 26.00]	16.00 [14.00 - 18.00]	17.00 [11.00 - 23.50]	8.6*10-154
**FAB**	14.00 [11.00 - 16.00]	14.00 [12.00 - 16.00]	13.00 [10.00 - 16.00]	8.00 [6.00 - 11.00]	6.00 [3.00 - 11.00]	3.6*10-88
**Short MNA**	10.00 [9.00 - 11.00]	11.00 [9.00 - 12.00]	10.00 [9.00 - 11.00]	9.00 [7.00 - 10.00]	7.00 [5.00 - 9.00]	4.2*10-57
**SPPB**	4.00 [2.00 - 6.00]	9.00 [8.00 - 10.00]	3.00 [1.00 - 5.00]	2.00 [1.00 - 3.00]	0.00 [0.00 - 1.00]	8.8*10-244
**Barthel scale**	85.00 [75.00 - 90.00]	95.00 [90.00 - 100.00]	85.00 [75.00 - 90.00]	70.00 [60.00 - 85.00]	25.00 [15.00 - 35.00]	3.1*10-168
**Lowton scale**	4.00 [3.00 - 5.00]	5.00 [4.00 - 7.00]	4.00 [3.00 - 5.00]	2.00 [1.00 - 4.00]	1.00 [0.00 - 1.00]	2.2*10-123
**GDS-5**	1.00 [0.00 - 2.00]	1.00 [0.00 - 1.00]	1.00 [0.00 - 2.25]	2.00 [1.00 - 3.00]	3.00 [2.00 - 4.00]	1.4*10-56
**Assistance in social and everyday living scale**	1.00 [0.00 - 2.00]	0.00 [0.00 - 1.00]	1.00 [0.00 - 2.00]	2.00 [1.00 - 4.12]	12.50 [6.00 - 18.00]	1.2*10-132
**Right hand grip strength**	13.00 [10.00 - 16.00]	13.00 [10.00 - 18.50]	13.00 [10.00 - 16.50]	10.00 [7.00 - 13.50]	7.50 [4.00 - 11.50]	4.9*10-46
**BMI**	25.50 [23.40 - 28.30]	24.80 [23.10 - 27.60]	26.60 [24.20 - 30.40]	25.15 [22.92 - 27.70]	24.20 [21.00 - 27.50]	1.7*10-8

*Note*. MMSE: mini-mental state examination; FAB: frontal assessment battery; MNA: mini nutritional assessment; SPPB: short physical performance battery; GDS-5: geriatric depression scale, version 5; BMI: body mass index.

### Validation of the aging phenotype calculator

The APC was validated on a sample of 96 older adults recruited in 2023, following the established protocol. The APC calculated the probability of each of the five aging phenotypes for every participant. The APC assigned to each participant the phenotype with the highest probability.

The aforementioned data were shared with geriatricians from both outpatient and inpatient departments of the Russian Clinical Research Center for Gerontology. Their task was to determine the aging phenotype of each participant. To evaluate the accuracy, precision, recall, and F-score, their conclusions were compared with the results produced by the APC. Additionally, ROC curves were generated using the OvR strategy.

## RESULTS

### Clustering

The initial step of clustering generated two distinct clusters that significantly differed in all the above-mentioned indicators (Supplementary Fig. 1A). The larger cluster was further clustered (see the Methods section), which generated four additional clusters (see Supplementary Fig. 1B). To facilitate comparison, each of the five obtained clusters was assigned a number: 0, 1, 2, 3, and 4 (Supplementary Fig. 1).

### Cluster description

**Figure 2.**
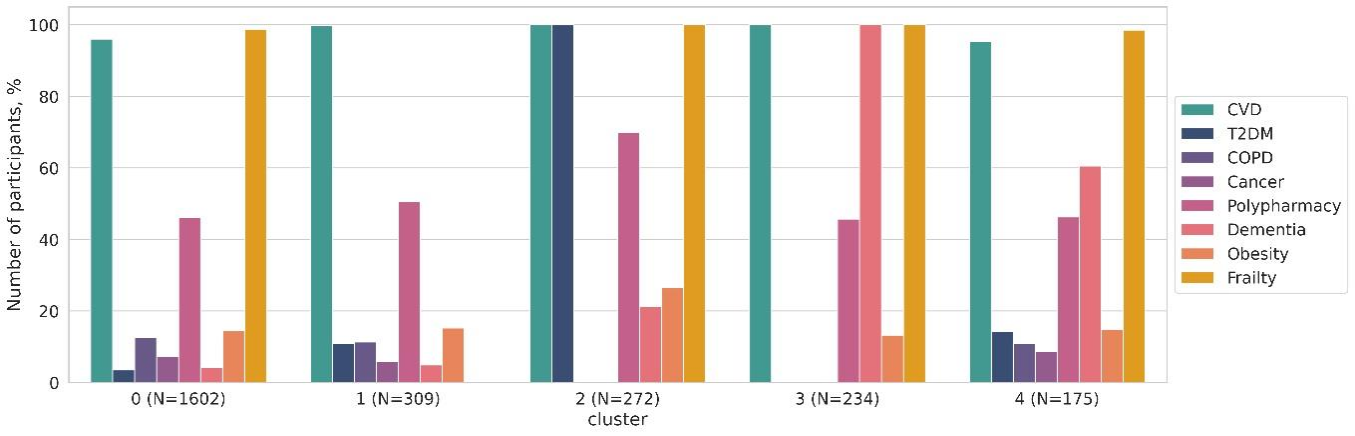
**Aging-associated diseases in Clusters 0, 1, 2, 3, and 4**. *Note: CVD: cardiovascular diseases; DM2: type 2 diabetes mellitus; COPD: chronic obstructive pulmonary disease.

The resulting clusters were compared based on the parameters reflecting the participants’ current health condition. Figure 2 shows the aging-associated diseases in each cluster. Based on the data shown in Figure 2 and the geriatric assessment results (Table 1), the clusters are classified as follows: Cluster 0, multimorbid frailty; Cluster 1, non-frailty; Cluster 2, metabolic frailty; Cluster 3, cognitive frailty; and Cluster 4, functional frailty.

**Figure 3.**
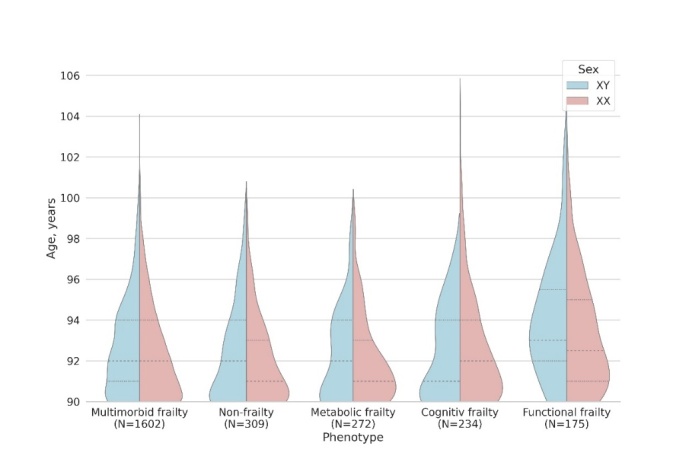
Sex and age composition in each cluster.

The clusters were compared based on their sex and age composition. The percentage of women was 59.9% in the non-frailty cluster, 74.5% in the multimorbid frailty cluster, 80.1% in the metabolic frailty cluster, 84.6% in the cognitive frailty cluster, and 82.3% in the functional frailty cluster. Pairwise comparisons (the chi-squared test) showed that the non-frailty cluster significantly differed from the multimorbid, metabolic, cognitive, and functional clusters with the following p-values: p-value = 1.4*10^-4^, p-value = 5.5*10^-5^, p-value = 8.7*10^-8^, and p-value = 1.2*10^-4^, respectively. Expectedly, participants in the functional frailty cluster were significantly older (Fig. 3) than in other clusters, except for the cognitive frailty cluster, with the following p-values of the difference from non-frailty, multimorbid frailty, metabolic frailty, and cognitive frailty: p-value =2.1*10^-4^, p-value =0.016, 3.8 * 10^-4^, and p-value = 0.36, respectively. Other pairwise comparisons did not show significant differences.

**Table 2:** The APC validation results.

Geriatrician 1					
**Phenotype**	Multimorbid	Non-frail	Metabolic	Cognitive	Functional
**Accuracy**	0.82	0.91	0.95	0.93	0.96
**Precision**	0.8	0.67	0.78	0.78	1
**Recall**	0.93	0.62	0.7	0.58	0.33
**F-score**	0.86	0.64	0.74	0.67	0.5
**Geriatrician 2**					
**Phenotype**	Multimorbid	Non-frail	Metabolic	Cognitive	Functional
**Accuracy**	0.93	0.97	0.98	0.94	0.98
**Precision**	0.89	1	0.89	0.89	0.5
**Recall**	1	0.8	0.89	0.62	0.5
**F-score**	0.94	0.89	0.89	0.73	0.5

**Figure 4.**
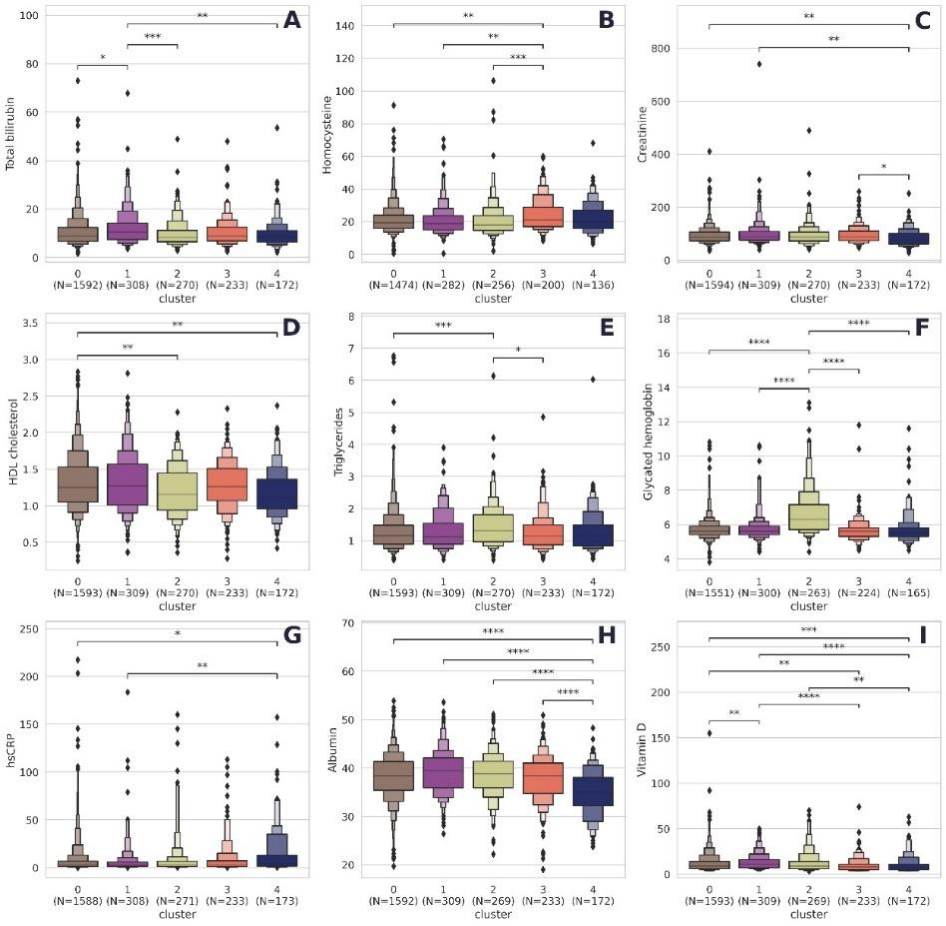
**Biochemical indicators in each cluster**. *Note*. *: 0.01< p <= 0.05; **; 0.001 < p <= 0.01; ***: 0.0001 < p <= 0.001; ****: p <= 0.0001. All p-values were obtained using the Mann-Whitney test and adjusted using the Bonferroni correction for multiple testing. Cluster 0, multimorbid frailty; Cluster 1, non-frail; Cluster 2, metabolic frailty; Cluster 3, cognitive frailty; Cluster 4, functional frailty.

The phenotypes also differed in the levels of biochemical indicators (Fig. 4) and other parameters (Supplementary Tables 1 and 2). Notably, glucose and glycated hemoglobin levels were significantly elevated in the metabolic frailty cluster (Fig. 4F; Supplementary Tables 1 and S). The levels of creatinine and albumin were considerably lower in the functional frailty cluster (Fig. 4C and 4H; Supplementary Tables 1 and 2).

Homocysteine levels were higher in the cognitive frailty cluster than in the non-frailty, multimorbid frailty, and metabolic frailty clusters (Fig. 4B; Supplementary Tables 1 and 2). Although total bilirubin levels were notably higher in the non-frailty cluster (Fig. 4A), they remained within the normal range across all clusters. High-sensitivity C-reactive protein (hsCRP) levels were higher in the functional frailty cluster (Fig. 4G). The levels of hsCRP in Clusters 0-3 were in the average range, i.e., above normal. The median hsCRP level in Cluster 4 increased to over 3 mg/l, which is typical for CAD and chronic illnesses if left untreated.

### Risk factors

Additional factors related to specific aging phenotypes were also evaluated. The APOE genotypes are recognized indicators of aging-associated diseases (Supplementary Tables 3 and 4). Having just one APOE-ε4 variant nearly doubled the likelihood of cognitive frailty (OR = 1. 9, p-value = 0. 01). No statistically significant associations were found for other clusters or the APOE ε2 variant.

The results of the analysis of the socioeconomic backgrounds and demographic indicators are shown in Supplementary Tables 5 and 6. The chi-square test showed statistically significant associations between the clusters and the place of residence (urban or rural), education level, occupation type (intellectual or physical), peak earnings, and life-long hobby. Gender- and age-adjusted logistic regression (Supplementary Table 6) was used to determine which factors were strongly associated with each cluster. Functional frailty was associated with a lifetime residence: urban residents were less likely to be functionally frail (OR = 0.34; p-value = 6.2*10^-4^). Education played a significant role in cognitive frailty, with even unfinished higher education (college or university) reducing the risk of cognitive impairment by half (OR = 0.58; p-value = 0.013). Having a life-long hobby had a similar effect (OR = 0.53; p-value = 0.006). Former high-income individuals were 72% more likely to be non-frail (OR = 1.72; p-value = 0.041), while former low-income individuals were more likely to be functionally frail (OR = 2.65; p-value = 7*10^-4^).

**Figure 5.**
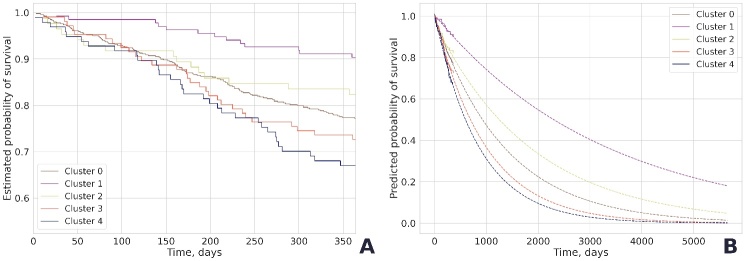
**The one-year survival rates in Clusters 0-4: real data (**A**); extrapolated data (**B**)**. Cluster 0, multimorbid frailty; Cluster 1, non-frailty; Cluster 2, metabolic frailty; Cluster 3, cognitive frailty; Cluster 4, functional frailty.

### Survival analysis

The one-year mortality was known for 962 participants. The survival curves for Clusters 0-4 (Fig. 5) differed significantly (p-value = 1.4*10^-6^). Expectedly, the highest survival rate (90.4%) was characteristic of the non-frailty cluster. All frailty clusters had similar survival dynamics in the first 100-110 days after enrollment. By the end of the observation period, the differences became more noticeable: 82.4% of older adults with metabolic frailty, 77.4% with multimorbid frailty, 72.6% with cognitive frailty, and 67% with functional frailty survived up to 365 days after enrollment.

The coefficients of determination reflecting the accuracy of the approximation of the exponential functions were 0.899 for non-frailty (Cluster 1), 0.996 for multimorbid frailty (Cluster 0), 0.953 for metabolic frailty (Cluster 2), 0.978 for cognitive frailty (Cluster 3), and 0.949 for functional frailty (Cluster 4). The predicted survival rates based on the extrapolated data were 2,300 days for non-frailty, 1,249 days for metabolic frailty, 926 days for multimorbid frailty, 694 days for cognitive frailty, and 597 days for functional frailty.

**Figure 6.**
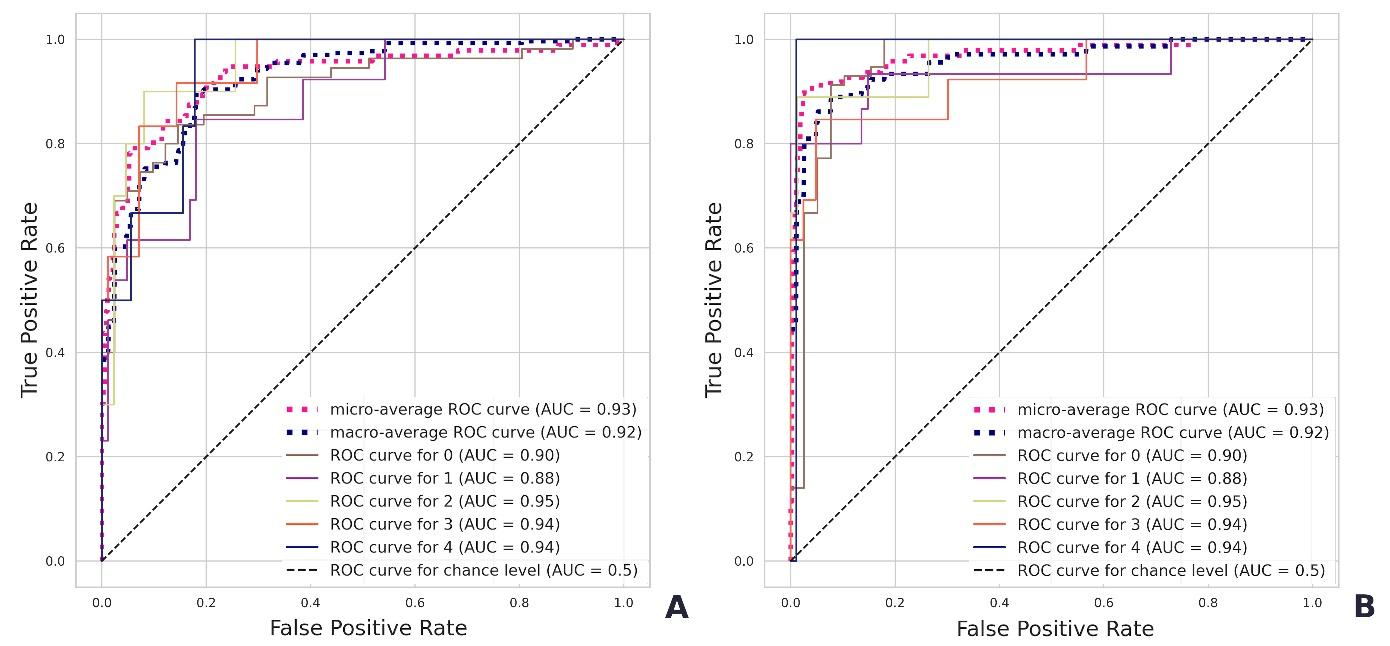
Quality of the validation sample classification by Geriatrician 1 (**A**) and Geriatrician 2 (**B**).

### Aging phenotype calculator

The APC was trained on 90% of the clustered data. The APC testing on the remaining 10% of the data generated an average ROC AUC of 0.9992. The OvR-based ROC AUC was 1.0 for non-frailty, 0.9996 for multimorbid frailty, 1.0 for metabolic frailty, 0.9999 for cognitive frailty, and 0.9967 for functional frailty (Supplementary Fig. 2).

For validation, the phenotype of 96 additionally recruited older adults was determined by geriatricians (Table 2, Fig. 6). The error matrices are shown in Supplementary Figures 3 and 4. The average ROC AUC of phenotyping was 0.923 for Geriatrician 1 (Fig. 6A) and 0.954 for Geriatrician 2 (Fig. 6B).

## DISCUSSION

In this study, we focused on frailty to demonstrate how comorbidities cause variations in aging phenotypes. The targeted clustering approach enabled the identification of five clinically significant aging phenotypes: non-frailty, multimorbid frailty, metabolic frailty, cognitive frailty, and functional frailty. The assessment of the APC's accuracy allowed us to recommend it as a clinically applicable geriatric decision-making tool for geriatric medicine.

### Non-frailty

People exhibiting no signs of frailty (the main criteria) were assigned to the non-frailty phenotype (Cluster 1). These are highly functional individuals with no diagnosed illnesses who score highly on all geriatric scales. However, a small percentage of them may develop health conditions, such as COPD, cancer, type 2 diabetes, and even dementia. Overall, 96% of the entire cohort had CVDs, suggesting a high prevalence of these diseases even in the non-frailty phenotype. Despite CVDs, non-frail older adults are independent and do not require assistance in daily living. Another important criterion for non-frailty is good performance on the Barthel scale (ADL, over 85 points) and the Lowton scale (IADL, a minimum of 4 points). Expectedly, the non-frail phenotype had the highest survival rate (median = 2,300 days from the assessment date).

There was a relatively high percentage of men in the non-frail cluster. Women are generally more likely to live to the age of 90. However, they tend to suffer from functional decline, whereas their male counterparts tend to stay healthier [7].

### Multimorbid frailty

The multimorbid frailty phenotype (Cluster 0) also included highly functional individuals. However, people with this aging phenotype show signs of frailty (SPPB < 8). The prevalence of CVDs is also high. Some of them have COPD, cancer, diabetes mellitus, and dementia. Interestingly, the multimorbid frail cluster was the largest in our study. Aging and age-related chronic diseases are underlain by the same mechanisms; hence, multimorbidity is widely viewed as a marker of aging [8].

Multimorbidity refers to the presence of two or more health conditions that may not be causally related. It implies the coexistence of multiple diseases rather than one underlying disease. Studies have shown that some populations are highly susceptible to multimorbidity, while others appear to be very resilient. Some disease comorbidities may not be accidental. A well-known example of the comorbidity of chronic conditions is COPD. It occurs in people with systemic inflammatory response syndrome and is associated with the progressive development of atherosclerosis [9], osteoporosis [10], chronic kidney disease [11], and lung cancer [12].

Multimorbidity is associated with a poor prognosis and a high risk of disability [13]. In our study, the survival rate in the multimorbid frail phenotype was 2.5 times lower than in the non-frail phenotype, with the median survival rate decreasing to 926 days.

On the one hand, multimorbidity requires simultaneous treatment of each comorbid condition. On the other hand, such treatment may become cumbersome for the patient. For example, an 80-year-old woman with six chronic diseases (osteoporosis, osteoarthritis, type 2 diabetes, hypertension, dyslipidemia, and COPD) may be prescribed 12 medications to be taken five times a day. Polypharmacy increases the risk of side effects from drug interactions. This results in a so-called prescribing cascade when the side effects of a drug are mistaken for a new disease, leading to additional prescriptions.

Given the challenges of managing patients with multimorbid frailty, an individualized patient-specific management approach should be adopted, prioritizing the disease that needs to be treated first to improve the quality of the patient’s life and health prognosis. At the same time, it is necessary to de-prescribe medications and discontinue those offering limited benefits or having side effects.

### Metabolic frailty

The main characteristic of people with the metabolic frailty phenotype (Cluster 2) is type 2 diabetes, in addition to CVDs and frailty. The metabolic phenotype is similar to the metabolic syndrome, which is associated with an increased risk of simultaneously developing severe arterial hypertension (AH), diabetes mellitus, lipid metabolism disorders, and ischemic heart disease [14, 15]. Interestingly, older adults in this cluster often had dementia as a comorbidity. Many studies have reported the comorbidity of dementia and diabetes mellitus [16-18].

As expected, metabolically frail participants had a higher BMI and were more obese. They also had increased levels of carbohydrate metabolism indicators, such as glycated hemoglobin and glucose. Although this biomarker was not an inclusion criterion, all participants with a glycated hemoglobin level of over 6% were assigned to the metabolic phenotype.

Despite the known correlation between frailty and metabolic syndrome in older people [19], participants with the metabolic phenotype in our study did not show serious functional decline. The survival rates in these participants were the highest of all the frailty groups, with a median survival rate of 1,249 days.

The treatment of metabolically frail people should also be individualized due to possible comorbidities. Managing carbohydrate metabolism in these individuals may not be the best solution for improving their quality of life. Greenfield et al. showed that in patients with type 2 diabetes and comorbidities, intensive glucose-lowering therapy is less effective in decreasing the risk of CVDs, and other options should be considered to improve the patient’s prognosis [20]. The metabolic phenotype should be managed, among other things, with lifestyle changes and the adoption of a non-pharmacological approach as a viable alternative to drug therapy.

### Cognitive frailty

Importantly, all clusters in our study included some proportion of older adults with cognitive impairment. Older individuals with cognitive impairment who also suffered from diabetes were assigned to the metabolic phenotype. Those who had cognitive impairment without signs of frailty were assigned to the non-frail phenotype. The cognitive phenotype (Cluster 3) included only those who had cognitive impairment as an independent disease that was not associated with other geriatric syndromes.

Cognitively frail participants had increased homocysteine levels, which is consistent with the consensus statement made by a group of researchers in 2018 [21]. Managing homocysteine levels may reduce the risk of cognitive impairment in older people. Treatment of patients with cognitive frailty should incorporate medical therapy and other treatment options. It should also address other non-cognitive neuropsychiatric disorders (behavioral, psychotic, affective, etc.). A critical step in the treatment of the cognitive phenotype, as well as functional frailty, would be a new paradigm of medical care, including the establishment of specialized facilities, such as memory rooms and clinics, geriatric psychological and social aid offices, boarding houses for individuals with cognitive impairment, and other solutions.

Cognitive stimulation is essential for cognitively frail patients—it helps them tap into their cognitive potential. Many cognitive stimulation techniques have been proposed, such as cognitive training and neurointerfaces. Cognitive training consists of attention, concertation, and focus exercises, multisensory interactions, and mnemonics [22, 23]. Neurointerfaces immerse users in sensory-stimulating virtual environments [24]. Virtual cognitive training has been shown to improve not only cognitive functions but also emotional well-being and daily functioning [24, 25].

### Functional frailty

The functional frailty phenotype (Cluster 4) includes people with severe frailty (SPPB < 2) who are in critical need of daily assistance. Interestingly, the participants with this aging phenotype had increased levels of ferritin in their blood (Supplementary Table 1). Previously, higher ferritin levels, much higher than in our study, have been associated with an increased risk of mortality in different populations [26]. They also had increased hsCRP levels (Fig. 4G), which indicates chronic inflammation and, as expected in functionally frail individuals, a large number of aging-associated diseases. However, it is important to note that participants with other aging phenotypes also had above-normal hsCRP levels. The survival rate in the functionally frail phenotype was, expectedly, much lower, with a median survival rate of less than two years. Participants with the functional frailty phenotype were older than those with other phenotypes. It is safe to assume that all of the above aging phenotypes eventually progress into the functional frailty phenotype. However, this hypothesis requires a longitudinal study.

Life expectancy is a key parameter in selecting the best treatment for functionally frail patients. It should be balanced against the priorities (survival, autonomy, pain and symptom relief, and palliative care needs) of patients and their relatives or caregivers. The expected benefits must be carefully weighed against possible disadvantages and side effects. Prognosis is primarily determined by the degree of functional dependence and the severity of physical, cognitive, biological, and social impairment. Given the diversity of issues, the paradigm of medical care should include collaboration between multiple services, such as social services, long-term care institutions, nursing homes, boarding houses, palliative care facilities, and hospices.

Similar aging phenotypes have been described in other studies. Marcucci et al. identified four aging phenotypes in 2,841 patients 65 years of age and older [2]. Their healthiest phenotype may correspond to our non-frailty phenotype; multimorbidity to our multimorbid frailty phenotype; functional dependence with cognitive impairment to our cognitive frailty phenotype. The authors obtained similar mortality rates in participants with multimorbid and cognitive frailty, which were significantly higher than in healthy participants [2]. Bekić et al. divided 263 older participants at least 60 years of age into four groups: (1) highly functional, (2) cognitively impaired, (3) cognitively frail, and (4) physically frail [27]. Groups 1 and 2 would have been assigned to the non-frail phenotype in our study; Group 3 would have been assigned to the cognitively frail phenotype; and Group 4 would have included the rest. The differences in the identified aging phenotypes may primarily result from the differences in clustering techniques, key parameters, and participant recruitment criteria.

### Risk factors

Aging is affected by both genetic and non-genetic factors. Some gene variants are known to be associated with longevity. In our study, we assessed the frequency of the APOE alleles as an aging phenotype predictor. Our finding on the statistical significance of APOE ε4 for the cognitive frailty phenotype is consistent with the published data on its involvement in cognitive impairment, particularly neurodegenerative diseases such as Alzheimer's disease [28]. We have demonstrated this association in a sample of older adults [29].

However, genetic predisposition accounts for about 20-30% of the likelihood of longevity [30], whereas the remaining 70-80% is determined by non-hereditary factors, such as lifestyle, socio-economic background, education, etc. The cognitive frailty phenotype in our study had the smallest number of people with higher education (Supplementary Table 6). Even in participants with unfinished higher education, the risk of cognitive frailty was two times lower (Supplementary Table 7). This is mainly because the cognitive assessment in our study was based on MMSE scores, which consistently show an association with the level of education [31, 32]. The association between MMSE scores and education can be interpreted in two ways: 1) education contributes to the preservation of cognitive functions; 2) people with a lower level of education may not be able to understand the MMSE questions and, therefore, perform worse. Similarly, those who had a life-long hobby were two times less likely to be cognitively frail.

High earners at the peak of their professional career, who generally had a better quality of life, were 72% more likely to have the non-frail aging phenotype. Low income, on the contrary, increased the risk of multimorbidity and the functional frailty phenotype. People with a lower income may often be malnourished, which negatively affects their overall health. Despite their long lifespan (≥ 90 years), they are susceptible to many chronic diseases.

### Aging phenotype calculator and survival prediction

Despite numerous studies on aging phenotypes, their findings have not yet been effectively incorporated into geriatric healthcare. Attempts have been made to integrate various machine learning solutions into geriatric care not only for medical decision-making (e.g., decision-making algorithms used in cancer treatment [33, 34]) but also for improving the quality of patients’ lives. Accelerometers built into wearable devices can feed into an algorithm that notifies physicians or caregivers of patients with neurological disorders about frequent falls or other critical changes in their functioning [35].

Quality of life questionnaires have been used in combination with neural networks that factor in all parameters simultaneously [36].

The Aging Phenotype Calculator presented in this study enables assessing the current functional status of older adults based on the results of their examination and geriatric assessment. It calculates the probability of each of the five aging phenotypes. Using the generated data and practical recommendations, coupled with their professional experience, geriatricians can make well-informed decisions about appropriate management or treatment strategies. The aging phenotype calculator may serve as the basis of a comprehensive machine learning-based geriatric decision-making system.

### Limitations

The main limitation of this study is the lack of long-term follow-up. With a one-year follow-up, we could not obtain reliable data on long-term survival and had to mathematically extrapolate the survival curves. Moreover, the identified aging phenotypes reflect the current state and do not take into account aging dynamics. Aging phenotypes are specific to the time of the assessment and may change over time.

### Conclusion

Geriatric decision-making entails a complex process of weighing out treatment options against patients’ priorities, such as quality of life and survival. The aging phenotype reflects the specific aging pattern characteristic of an individual. It serves as an instructive basis for health management and treatment prioritization. Here, we present five aging phenotypes found in a sample of long-living adults: non-frailty, multimorbid frailty, metabolic frailty, cognitive frailty, and functional frailty. Each of the five aging phenotypes is an indicator of the underlying processes with distinct clinical manifestations that require specialized patient management. The aging phenotype also affects the survival of older adults. Non-frail older adults have the highest survival rate, whereas functionally frail older adults have the lowest survival rate. This indicates that establishing the aging phenotype of a patient may assist geriatricians in personalizing and optimizing treatment or management strategies and improving quality of life. The presented machine-learning-based calculator estimates the aging phenotype with an accuracy of 92% and may simplify geriatric decision-making. The calculator provides an objective and accurate assessment of the patient's aging phenotype. This offers two major benefits. The calculator can be used to streamline prioritizing options for preventative care, lifestyle changes, and medical interventions, which leads to more individualized, strategic, and effective healthcare for older individuals. It also enables efficient and reliable patient stratification, which is critical both for geriatric medical practice and for more focused fundamental research into the phenomena of longevity and successful aging.

### Ethical considerations

The study was approved by the Local Ethics Committee of the Russian Gerontology Research and Clinical Center (Protocol No. 30 from December 24, 2019).

## Supplementary Materials



## Author Statement

All authors approved the manuscript and gave their consent for submission and publication.
