# Supplementary material for: Machine Learning-Based Decision-Making in Geriatrics: Aging Phenotype Calculator and Survival Prognosis
Source: Aging Dis. 2024 Jan 20;16(1):565–77. doi: 10.14336/AD.2024.0120 (PMC11745442; doi:10.14336/AD.2024.0120)
Supplement: Supplementary file 1 [file AD-16-1-565-s.pdf]

SUPPLEMENTARY DATA

Machine Learning-Based Decision-Making in Geriatrics: Aging Phenotype Calculator and Survival Prognosis

Aleksandra Mamchur, Natalia Sharashkina, Veronika Erema, Daria Kashtanova, Mikhail Ivanov, Maria Bruttan, Elena Zelenova, Eva Shelly, Valentina Ostapenko, Irina Dzhumaniiazova, Lorena Matkava, Vladimir Yudin, Anna Akopyan, Irina Strazhesko, Lilit Maytesyan, Irina Tarasova, Olga Beloshevskaya, Anton Keskinov, Sergey Kraevoy, Olga Tkacheva, Sergey Yudin

© 2024. Yang J et al. Published online at http://www.aginganddisease.org/EN/10.14336/AD.2024.0303

SUPPLEMENTARY DATA

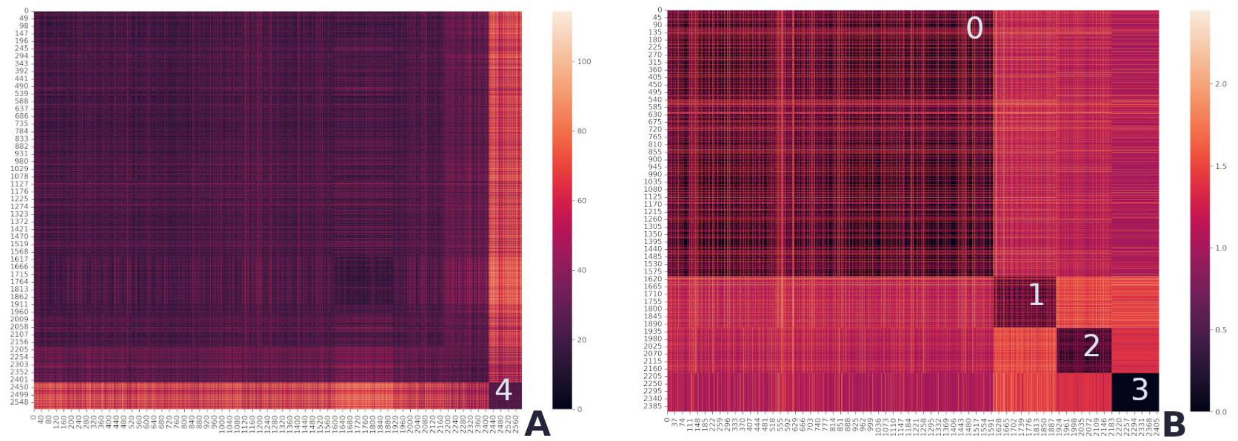

Supplementary Figure 1. Euclidean distance matrix for the first (A) and second (B) clustering steps.

Supplementary Table 1. Physical examination and laboratory testing results in different aging phenotypes (only significant).

**Cluster**	**0**	**1**	**2**	**3**	**4**	**p-value (Kruskal test)**
**Phenotype**	**Multimorbid frailty (n=1602)**	**Non-frail (n=309)**	**Metabolic frailty (N=272)**	**Cognitive frailty (N=234)**	**Functional frailty (n=175)**	
**Indicator**	Me [Q1-Q3]	Me [Q1-Q3]	Me [Q1-Q3]	Me [Q1-Q3]	Me [Q1-Q3]	
**Current health indicators**						
**Right hand grip strength**	13.00 [10.00 - 16.00]	13.00 [10.00 - 18.50]	13.00 [10.00 - 16.50]	10.00 [7.00 - 13.50]	7.50 [4.00 - 11.50]	$4.9*10^{-46}$
**Left hand grip strength**	12.00 [9.00 - 15.05]	13.50 [9.00 - 18.00]	12.00 [9.00 - 16.00]	9.00 [7.00 - 12.00]	6.25 [3.50 - 10.00]	$1.3*10^{-52}$
**Systolic blood pressure**	135.00 [126.00 - 146.00]	138.00 [127.00 - 150.00]	135.50 [127.00 - 148.75]	130.00 [120.00 - 150.00]	130.00 [110.00 - 140.00]	$3.1*10^{-5}$
**BMI**	25.50 [23.40 - 28.30]	24.80 [23.10 - 27.60]	26.60 [24.20 - 30.40]	25.15 [22.92 - 27.70]	24.20 [21.00 - 27.50]	$1.7*10^{-8}$
**Waist circumference**	92.00 [86.00 - 98.00]	92.00 [82.00 - 98.00]	95.00 [87.00 - 103.00]	89.50 [80.00 - 98.00]	90.00 [79.00 - 98.00]	$2.1*10^{-5}$
**13B-T (total bilirubin)**	8.90 [6.67 - 12.40]	10.50 [7.45 - 14.22]	8.25 [6.50 - 11.17]	8.90 [6.90 - 12.60]	8.30 [6.40 - 11.15]	$6.8*10^{-4}$
**1631NTP (NT-proBNP)**	621.50 [310.00 - 1489.00]	507.00 [256.00 - 1093.00]	546.50 [302.00 - 1130.75]	740.00 [390.00 - 1669.00]	787.00 [368.50 - 1774.75]	0.007
**16GLU (glucose)**	5.00 [4.50 - 5.60]	5.10 [4.60 - 5.70]	5.80 [5.00 - 7.57]	5.10 [4.70 - 5.70]	4.90 [4.50 - 5.70]	$2.4*10^{-20}$
**172INS (insulin)**	6.60 [4.60 - 10.17]	7.20 [4.90 - 11.10]	7.20 [4.93 - 11.53]	6.60 [4.40 - 11.20]	5.15 [3.60 - 8.72]	$2.9*10^{-4}$
**174S-C (IGF-1)**	99.40 [77.93 - 131.55]	105.75 [81.80 - 136.03]	109.80 [88.10 - 144.20]	99.70 [78.62 - 133.38]	90.85 [65.58 - 124.75]	$2.6*10^{-4}$
**18HBA1 (HbA1c (glycated haemoglobin))**	5.60 [5.40 - 5.90]	5.60 [5.40 - 5.90]	6.30 [5.70 - 7.15]	5.60 [5.30 - 5.80]	5.50 [5.30 - 5.80]	$2.8*10^{-44}$
**22CRE (creatinine)**	86.00 [72.25 - 105.75]	88.00 [75.00 - 108.00]	87.00 [72.00 - 105.75]	88.00 [73.00 - 109.00]	76.00 [61.00 - 100.25]	0.005

© 2024. Yang J et al. Published online at http://www.aginganddisease.org/EN/10.14336/AD.2024.0303

**28TP (total protein)**	69.00 [64.00 - 74.00]	69.00 [65.00 - 74.00]	70.00 [64.00 - 74.00]	69.00 [66.00 - 74.00]	67.00 [61.00 - 71.00]	0.03
**29A1 (alpha-1-globulin)**	3.10 [2.80 - 3.50]	3.00 [2.70 - 3.30]	3.10 [2.80 - 3.50]	3.20 [2.80 - 3.60]	3.30 [3.00 - 3.70]	$3.9*10^{-5}$
**30TG (triglycerides)**	1.15 [0.89 - 1.48]	1.12 [0.89 - 1.54]	1.31 [0.96 - 1.81]	1.14 [0.87 - 1.49]	1.16 [0.84 - 1.49]	0.004
**32HDL (HDL)**	1.25 [1.05 - 1.53]	1.27 [1.01 - 1.57]	1.15 [0.94 - 1.45]	1.26 [1.07 - 1.51]	1.11 [0.96 - 1.36]	$1.3*10^{-5}$
**51FERR (ferritin)**	62.00 [32.00 - 112.50]	70.00 [36.50 - 121.00]	60.00 [30.00 - 115.00]	65.50 [34.25 - 118.50]	97.00 [41.00 - 175.00]	0.037
**53FT3 (free T3)**	3.60 [3.20 - 4.00]	3.70 [3.30 - 4.00]	3.50 [3.10 - 3.80]	3.60 [3.20 - 3.90]	3.40 [2.90 - 3.80]	$1.2*10^{-5}$
**5PLT (platelets)**	220.00 [183.00 - 266.75]	205.00 [171.75 - 244.50]	219.00 [177.00 - 278.00]	225.00 [184.00 - 279.00]	226.00 [181.00 - 274.00]	0.025
**8ALT (alanine transaminase)**	11.00 [9.00 - 15.00]	12.00 [10.00 - 16.00]	12.00 [9.00 - 16.00]	11.00 [8.00 - 15.00]	9.50 [7.00 - 14.00]	$1*10^{-4}$
**928VD3 (25(OH) D, vitamin D)**	9.00 [6.00 - 14.00]	11.00 [7.00 - 16.00]	9.00 [6.00 - 14.00]	8.00 [5.00 - 11.00]	7.00 [5.00 - 11.00]	$5.1*10^{-12}$
**9AST (aspartate aminotransferase)**	18.00 [15.00 - 21.00]	18.00 [16.00 - 21.00]	16.00 [14.00 - 20.00]	17.00 [15.00 - 22.00]	16.00 [14.00 - 19.00]	$4.6*10^{-6}$
**ALB-PF (albumin)**	38.40 [35.40 - 41.30]	39.50 [35.90 - 42.10]	38.80 [35.90 - 41.40]	38.40 [34.70 - 41.00]	35.00 [32.18 - 38.00]	$3.8*10^{-17}$
**ALB/CRU (urine albumin-creatinine ratio)**	22.00 [12.00 - 45.75]	23.00 [11.00 - 47.50]	34.00 [16.00 - 67.50]	31.00 [14.00 - 62.00]	35.00 [16.00 - 82.00]	0.007
**hsCRP**	2.69 [1.27 - 6.85]	2.29 [1.14 – 6.04]	2.9 [1.29 – 6.65]	2.59 [1.11 – 7.19]	4.19 [1.64 – 12.83]	0,044

Supplementary Table 2. Pairwise phenotype comparison

Cluster	0VS1	0VS2	0VS3	0VS4	1VS2	1VS3	1VS4	2VS3	2VS4	3VS4
**Indicator**	p-value of pairwise comparisons (Mann–Whitney U test) adjusted for multiple testing									
**MMSE**	**$1.3*10^{-8}$**	0.026	**$2.3*10^{-121}$**	**$1.5*10^{-36}$**	**$1.8*10^{-10}$**	**$5.2*10^{-77}$**	**$2.9*10^{-34}$**	**$1.1*10^{-48}$**	**$3.7*10^{-15}$**	1
**FAB**	1	1	**$8.6*10^{-52}$**	**$2.7*10^{-42}$**	0.36	**$5.7*10^{-38}$**	**$4.5*10^{-32}$**	**$6.7*10^{-24}$**	**$1.5*10^{-22}$**	1
**MNA**	0.77	1	**$1.1*10^{-15}$**	**$5.4*10^{-41}$**	1	**$2.3*10^{-16}$**	**$2.0*10^{-33}$**	**$8.9*10^{-9}$**	**$1.7*10^{-27}$**	**$6*10^{-9}$**
**Right hand hand grip strength**	1	1	**$1.6*10^{-14}$**	**$1.2*10^{-34}$**	1	**$2.8*10^{-10}$**	**$5.4*10^{-25}$**	**$4.2*10^{-7}$**	**$5.5*10^{-21}$**	**$2.4*10^{-6}$**
**Left hand hand grip strength**	1	1	**$8.5*10^{-16}$**	**$5.7*10^{-38}$**	1	**$4.1*10^{-14}$**	**$1.5*10^{-30}$**	**$2.3*10^{-7}$**	**$6.1*10^{-23}$**	**$1*10^{-7}$**
**SPPB**	**$5.6*10^{-164}$**	0.149	**$2.9*10^{-23}$**	**$2.2*10^{-74}$**	**$9.4*10^{-95}$**	**$1.6*10^{-87}$**	**$3.1*10^{-74}$**	**$6*10^{-7}$**	**$1.8*10^{-45}$**	**$2.1*10^{-25}$**
**Barthel**	**$4.4*10^{-43}$**	1	**$3.9*10^{-22}$**	**$2.2*10^{-103}$**	**$6.4*10^{-35}$**	**$1.7*10^{-50}$**	**$2.5*10^{-73}$**	**$1.4*10^{-8}$**	**$6.5*10^{-69}$**	**$9*10^{-64}$**
**IADL**	**$1.5*10^{-27}$**	1	**$1.6*10^{-21}$**	**$1.9*10^{-79}$**	**$3.4*10^{-20}$**	**$5*10^{-35}$**	**$4.3*10^{-64}$**	**$8*10^{-9}$**	**$2.6*10^{-47}$**	**$1.6*10^{-15}$**
**GDS-5**	0.617	1	**$6.5*10^{-18}$**	**$2.2*10^{-37}$**	**0.002**	**$6*10^{-21}$**	**$1.9*10^{-35}$**	**$2.4*10^{-5}$**	**$2.9*10^{-19}$**	**$5.5*10^{-5}$**

© 2024. Yang J et al. Published online at http://www.aginganddisease.org/EN/10.14336/AD.2024.0303

SUPPLEMENTARY DATA

**Assistance in social and everyday living scale**	**$1.5*10^{-18}$**	1	**$7.1*10^{-21}$**	**$7.4*10^{-95}$**	**$1.9*10^{-12}$**	**$1.3*10^{-34}$**	**$2.3*10^{-72}$**	**$5.3*10^{-10}$**	**$1.1*10^{-59}$**	**$7.2*10^{-42}$**
**Systolic blood pressure**	1	1	1	**$1*10^{-5}$**	1	1	**$3.6*10^{-5}$**	1	**0.001**	0.075
**Diastolic blood pressure**	1	1	1	0.193	1	1	0.339	1	0.186	**0.018**
**BMI**	1	**0.002**	1	**0.002**	**$5.6*10^{-4}$**	1	0.962	**0.007**	**$2.8*10^{-7}$**	1
**Waist circumference**	1	0.09	0.068	1	**0.047**	1	1	**$1*10^{-4}$**	0.125	1
**Total bilirubin**	**0.016**	1	1	1	**0.001**	1	**0.006**	1	1	1
**Homocysteine**	1	1	**0.01**	1	1	**0.006**	1	**$7.8*10^{-4}$**	1	1
**NT-proBNP**	1	1	1	1	1	**0.037**	0.157	0.546	1	1
**Glucose**	1	**$1.7*10^{-21}$**	1	1	**$4.2*10^{-9}$**	1	1	**$9.3*10^{-7}$**	**$3.2*10^{-8}$**	1
**Insulin**	1	1	1	**0.018**	1	1	**0.001**	1	**$2.5*10^{-4}$**	0.422
**IGF-1**	1	**0.027**	1	1	1	1	0.054	1	**0.001**	1
**HbA1c (glycated haemoglobin, Hb)**	1	**$5.5*10^{-41}$**	1	0.068	**$7.5*10^{-23}$**	1	0.104	**$5.3*10^{-24}$**	**$5*10^{-22}$**	1
**Creatinine**	1	1	1	**0.005**	1	1	**0.002**	1	0.185	**0.017**
**Total protein**	1	1	1	**0.024**	1	1	**0.011**	1	0.249	0.074
**alpha-1-globulin**	1	1	1	**0.028**	1	**0.012**	**$1.2*10^{-4}$**	1	0.145	1
**Triglycerides**	1	**$4.7*10^{-4}$**	1	1	0.312	1	1	**0.024**	0.607	1
**HDL**	1	**0.004**	1	**0.003**	0.149	1	0.067	1	1	0.466
**Ferritin**	1	1	1	**0.012**	1	1	1	1	0.114	1
**Free T3**	1	1	1	**0.001**	**0.03**	1	**$1.6*10^{-5}$**	1	1	0.069
**Platelets**	**0.038**	1	1	1	1	**0.020**	0.605	1	1	1
**Testosterone**	**$6.7*10^{-4}$**	1	1	1	**$1.5*10^{-5}$**	**0.043**	**$8.2*10^{-5}$**	1	1	1
**Alanine transaminase**	1	1	1	**0.004**	1	1	**$6.6*10^{-5}$**	1	**0.03**	1
**Vitamin D (25(OH) D)**	**0.004**	1	**0.01**	**$4*10^{-4}$**	1	**$1.1*10^{-7}$**	**$2.4*10^{-8}$**	0.091	**0.005**	1
**Albumin in the urine (concentration)**	1	1	1	0.14	1	1	0.843	1	1	1ju
**Aspartate aminotransferase**	1	**0.022**	1	**0.002**	**0.009**	1	**$3*10^{-4}$**	1	1	0.674
**Albumin**	0.665	1	1	**$2*10^{-14}$**	1	0.229	**$1.7*10^{-16}$**	1	**$2.8*10^{-12}$**	**$2.6*10^{-6}$**
**ALB/CRU (urine albumin-creatinine ratio)**	1	**0.047**	1	1	1	1	1	1	1	1
**hsCRP**	1	1	1	**0.025**	1	1	**0.0098**	1	0.93	0.49
**Monocytes, %**	1	0.095	1	**0.001**	**0.001**	0.127	**$8.9*10^{-6}$**	1	1	1

© 2024. Yang J et al. Published online at http://www.aginganddisease.org/EN/10.14336/AD.2024.0303

SUPPLEMENTARY DATA

Supplementary Table 3. Frequency of occurrence of the APOE genotypes

Genotype	Cluster 0 (1,602)	Cluster 1 (309)	Cluster 2 (272)	Cluster 3 (234)	Cluster 4 (175)	Entire sample	p-value (chi square)
ε2ε2	19 (1.2%)	6 (1.9%)	1 (0.4%)	3 (1.3%)	2 (1.1%)	31 (1.2%)	0.005
ε2ε3	228 (14.2%)	56 (18.1%)	40 (14.7%)	41 (17.5%)	33 (18.9%)	398 (15.3%)	
ε2ε4	18 (1.1%)	4 (1.3%)	3 (1.1%)	3 (1.3%)	5 (2.9%)	33 (1.3%)	
ε3ε3	1166 (72.5%)	218 (70.6%)	204 (75%)	144 (61.5%)	109 (62.3%)	1841 (70.8%)	
ε3ε4	165 (10.3%)	25 (8.1%)	23 (8.5%)	40 (1.3%)	26 (14.9%)	279 (10.7%)	
ε4ε4	6 (0.4%)	0 (-)	1 (0.4%)	3 (1.3%)	0 (-)	10 (0.4%)	

Supplementary Table 4. Results of logistic regression using APOE genotypes

Cluster	ε2		ε4	
	coef	p-value	coef	p-value
**0**	-0.2257	0.3	-0.1519	1
**1**	0.3008	0.47	-0.4823	0.2
**2**	-0.1286	1	-0.2805	1
**3**	0.1423	1	0.6885	0.001
**4**	0.2959	1	0.5721	0.07

Supplementary Table 5. Comparison of the socioeconomic backgrounds (only significant)

Cluster		0	1	2	3	4	p-value (chi square)
**Phenotype**		Multimorbid frailty (n=1,602)	Non-frail (n=309)	Metabolic frailty (n=272)	Cognitive frailty (n=234)	Functional frailty (n=175)	
**Параметр**		n (%)	n (%)	n (%)	n (%)	n (%)	
**Lifelong residence**	Rural	87 (5.7%)	19 (6.7%)	6 (2.3%)	14 (6.8%)	22 (15.8%)	3*10-4
	Urban	1418 (94.3%)	264 (93.3%)	253 (97.7%)	191 (93.2%)	117 (84.2%)	
**Education**	Primary school or unfinished primary school	94 (5.8%)	27 (8.7%)	18 (6.6%)	35 (15.2%)	23 (13.2%)	3.46*10-7
	Middle school or unfinished middle school	197 (12.3%)	23 (7.4%)	36 (13.2%)	36 (15.6%)	28 (16.1%)	
	Complete high school	271 (17%)	46 (14.9%)	48 (17.7%)	56 (24.2%)	32 (18.4%)	
	Vocational school	135 (8.4%)	25 (8.1%)	15 (5.5%)	13 (5.6%)	4 (2.3%)	
	Training school	278 (17.4%)	38 (12.3%)	44 (16.2%)	30 (13%)	27 (15.5%)	
	Undergraduate	23 (1.4%)	3 (1%)	6 (2.2%)	1 (0.4%)	4 (2.3%)	
	Graduate	557 (35%)	140 (45.3%)	93 (34.2%)	55 (23.8%)	53 (30.5%)	
	Postgraduate	43 (2.7%)	7 (2.3%)	12 (4.4%)	5 (2.2%)	3 (1.7%)	
**Occupation**	Primarily physical	370 (23.3%)	78 (25.6%)	62 (22.9%)	83 (35.8%)	54 (312%)	0.018
	Primarily intellectual	582 (36.7%)	120 (39.3%)	111 (41%)	71 (30.6%)	67 (39.6%)	
	Intellectual + physical	636 (40%)	107 (35.1%)	98 (36.1%)	78 (33.6%)	48 (28.4%)	
**Peak income**	Low	109 (7.2%)	26 (9.2%)	16 (6.2%)	32 (15.6%)	28 (20.3%)	1.6*10-9

© 2024. Yang J et al. Published online at http://www.aginganddisease.org/EN/10.14336/AD.2024.0303

	Medium	1235 (82%)	201 (71%)	211 (81.5%)	153 (74.6%)	87 (63%)	
	High	162 (10.8%)	56 (19.8%)	32 (12.3%)	20 (9.8%)	23 (16.7%)	
**Lifelong hobby**	No	832 (56.1%)	182 (65%)	129 (49.6%)	140 (69.3%)	79 (58.1%)	0.002
	Yes	652 (43.9%)	98 (35%)	131 (50.4%)	62 (30.7%)	57 (41.9%)	

Supplementary Table 6. Effects of the background and socioeconomic background on the aging phenotype (based on logistic regression adjusted for age and sex).

Indicator	Non-frailty		Multimorbid frailty		Metabolic frailty		Cognitive frailty		Functional frailty	
	OR	p-val	OR	p-val	OR	p-val	OR	p-val	OR	p-val
Residence	1.21	1	0.84	1	2.94	0.319	0.92	1	**0.34**	**6.2*10^{-4}**
Graduate/undegraduate	1.00	1	1.41	0.196	1.18	1	**0.58**	**0.013**	0.83	1
Intelelctual work	0.95	1	1.19	1	1.19	1	0.7	0.504	1.08	1
High peak income	0.69	0.095	**1.72**	**0.041**	1.11	1	0.83	1	1.54	1
Low peak income	**0.59**	**0.01**	1.27	1	0.62	1	1.9	0.064	**2.65**	**7*10^{-4}**
Lifelong hobby	1.19	1	0.75	0.983	1.44	0.172	**0.53**	**0.006**	0.91	1

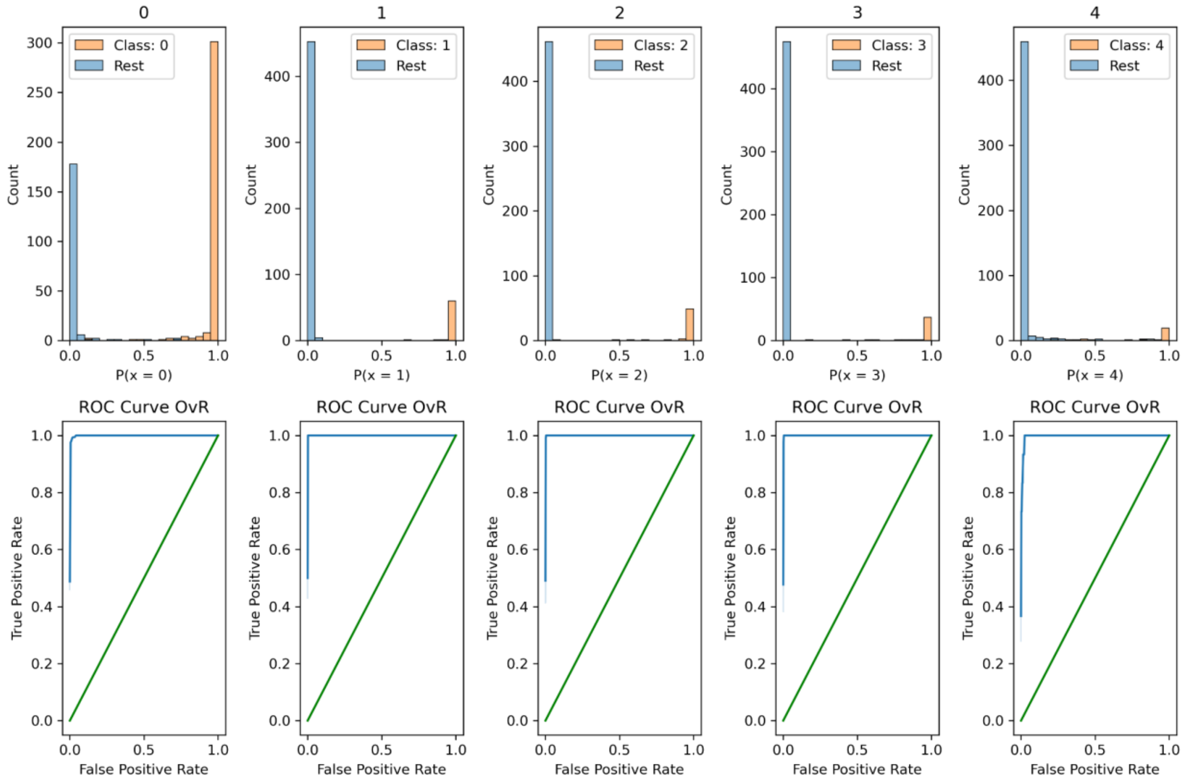

Supplementary Figure 2. Calculator quality assessment on the test set.

© 2024. Yang J et al. Published online at http://www.aginganddisease.org/EN/10.14336/AD.2024.0303

SUPPLEMENTARY DATA

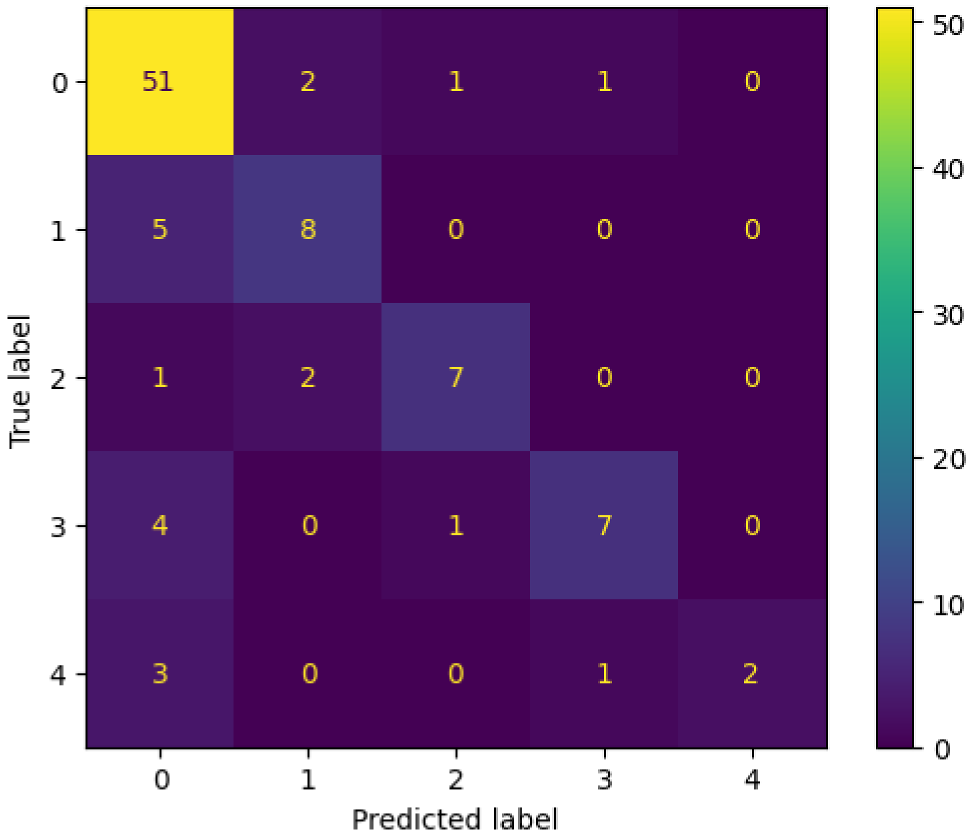

Supplementary Figure 3. Validation error matrix for geriatrician 1.

© 2024. Yang J et al. Published online at http://www.aginganddisease.org/EN/10.14336/AD.2024.0303

SUPPLEMENTARY DATA

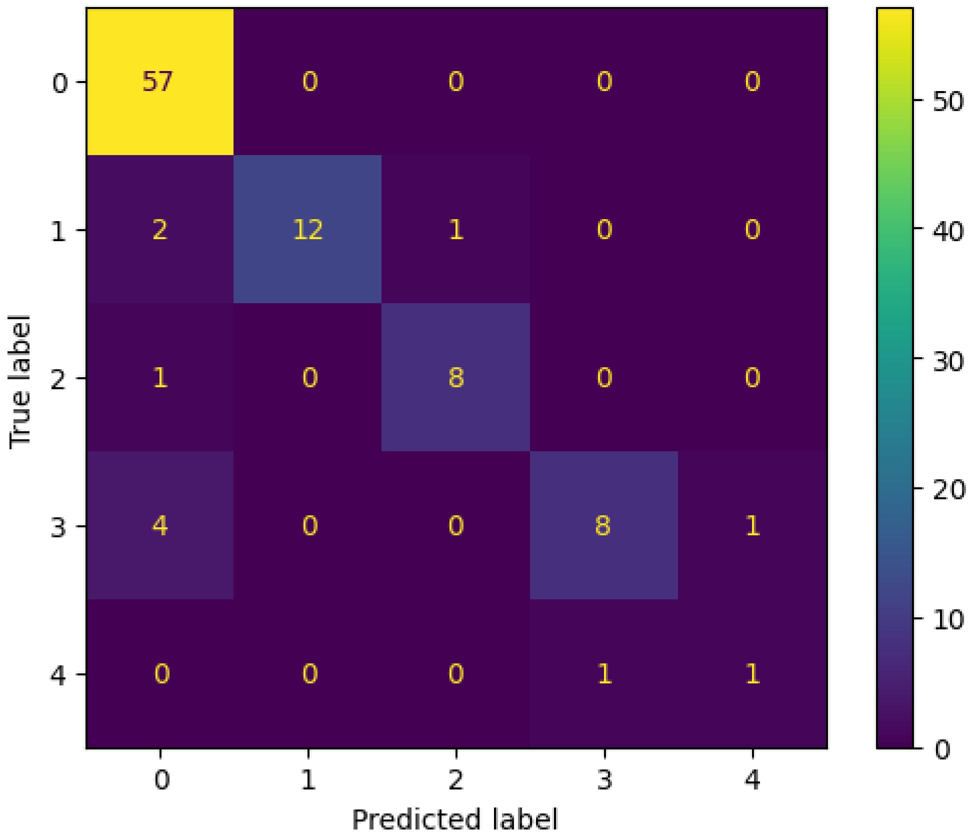

Supplementary Figure 4. Validation error matrix for geriatrician 2.

© 2024. Yang J et al. Published online at http://www.aginganddisease.org/EN/10.14336/AD.2024.0303
